# Ratiometric Upconversion Temperature Sensor Based on Cellulose Fibers Modified with Yttrium Fluoride Nanoparticles

**DOI:** 10.3390/nano12111926

**Published:** 2022-06-04

**Authors:** Małgorzata Skwierczyńska, Natalia Stopikowska, Piotr Kulpiński, Magdalena Kłonowska, Stefan Lis, Marcin Runowski

**Affiliations:** 1 Department of Rare Earths, Faculty of Chemistry, Adam Mickiewicz University, Uniwersytetu Poznańskiego 8, 61-614 Poznań, Poland; natalia.stopikowska@amu.edu.pl (N.S.); blis@amu.edu.pl (S.L.); mrunowsk@ull.edu.es (M.R.); 2 Department of Mechanical Engineering, Informatics and Chemistry of Polymer Materials, Faculty of Material Technologies and Textile Design, Lodz University of Technology, Żeromskiego 116, 90-924 Łódź, Poland; piotr.kulpinski@p.lodz.pl; 3 Department of Knitting Technology and Textile Machines, Faculty of Material Technologies and Textile Design, Lodz University of Technology, Żeromskiego 116, 90-924 Łódź, Poland; magdalena.klonowska@p.lodz.pl; 4 Departamento de Física, IMN and IUdEA, Universidad de La Laguna, Apdo. Correos 456, E-38200 La Laguna, Spain

**Keywords:** ratiometric temperature sensor, multifunctional cellulose fibers, wearable sensor, lanthanide ions

## Abstract

In this study, an optical thermometer based on regenerated cellulose fibers modified with YF_3_: 20% Yb^3+^, 2% Er^3+^ nanoparticles was developed. The presented sensor was fabricated by introducing YF_3_ nanoparticles into cellulose fibers during their formation by the so-called Lyocell process using N-methylmorpholine N-oxide as a direct solvent of cellulose. Under near-infrared excitation, the applied nanoparticles exhibited thermosensitive upconversion emission, which originated from the thermally coupled levels of Er^3+^ ions. The combination of cellulose fibers with upconversion nanoparticles resulted in a flexible thermometer that is resistant to environmental and electromagnetic interferences and allows precise and repeatable temperature measurements in the range of 298–362 K. The obtained fibers were used to produce a fabric that was successfully applied to determine human skin temperature, demonstrating its application potential in the field of wearable health monitoring devices and providing a promising alternative to thermometers based on conductive materials that are sensitive to electromagnetic fields.

## 1. Introduction

Flexible sensors have recently attracted the interest of researchers, especially in the area of wearable devices, due to their ability to be conformed, bent, or rolled into the desired shapes, which greatly reduces the size of the device and makes mass production more economical. Sensors based on cellulose, which is a well-known natural biopolymer, are of particular interest [1,2,3,4,5]. The main advantages of cellulose are its availability, biodegradability, and low price [4,5,6,7,8]. Another merit of cellulose is that it is relatively easy to process and has a high modification potential, allowing cellulose and its derivatives (film, fibers, or papers) to be endowed with new features (e.g., antibacterial, catalytic conductive, luminescent, plasmonic) [9,10,11,12]. This type of modified cellulosic material can be successfully used for the production of flexible wearable sensors for human health monitoring devices, as they combine the characteristics of the modifier with the flexibility, air, and moisture permeability of cellulose, which ensure high comfort of use of the sensor. Furthermore, cellulose (in the form of regenerated fibers) is one of the basic materials used in the textile industry, which significantly increases the applicability of cellulose-based sensors in wearable health monitoring devices.

One of the most important physiological parameters where monitoring is essential in wearable health monitoring devices is temperature [6,13]. Conventional methods of temperature determination such as volume expansion thermometers, thermocouples, or thermistors are often insufficient, because there is a need for fast, remote, and real-time measurements that provide high spatial and temperature resolution [6,14,15]. Additionally, in many cases (for example, in magnetic resonance imaging (MRI)), any electromagnetic interferences (EMI) or galvanic connections are highly undesirable, and thus other nonelectrical methods of temperature measurement are sought [6,16,17,18]. Therefore, optical temperature sensors (e.g., based on fiber Bragg gratings, interferometers, thermography, Raman scattering, or luminescence) have become increasingly popular in the past few years because they meet the above requirements for temperature measurement [16,17,18,19,20,21].

There are a vast number of articles on flexible sensors based on cellulose and conductive materials (metal nanoparticles, carbon nanotubes, graphene oxide, or polypyrrole) used for temperature determination [4,5,22]; however, they show disadvantages that are characteristic of the electronic thermometers mentioned earlier. Therefore, developing a cellulose-based sensor that exploits optical phenomena to determine temperature remains an urgent and challenging task. This type of thermometer, due to its small size, light weight, flexibility, resistance to EMI, and electrical safety has the potential to be used in the design of wearable devices for human health monitoring.

Among the various optical methods used to determine temperature, luminescence thermometry appears to be a very promising alternative [23,24,25,26,27,28]. Temperature sensors based on the phenomenon of luminescence exploit temperature-induced changes in the spectroscopic properties of phosphors, for instance: changes in the intensity or width of emission bands, the appearance of new bands in the emission spectrum, shortening of emission lifetimes, changes in the luminescence (fluorescence) intensity ratio—LIR (FIR) of two bands, spectral shifts in absorption/emission bands, and different efficiency of energy and charge transfer processes [23,24]. One of the key advantages of luminescence-based methods is their resistance to the aforementioned EMI, as they rely on measuring the emission spectrum of a previously excited sample rather than measuring the conductivity (or resistivity) of the material [29,30]. Typically, luminescence-based temperature sensors use the LIR method for temperature determination [24,25]. These luminescent thermometers can be classified according to different standards, such as the energy conversion path (down-conversion or up-conversion), size (nano or micro), type of dopant ions (lanthanides or d-block metal ions), or number of emission centers (single or dual emission centers). Very popular types of LIR-based thermometers are those based on lanthanide ions (especially single-emission-centers type), due to their narrow emission bands derived from 4f-4f transitions and their high luminescence efficiency over a wide temperature range [31]. Changes in the emission spectrum of lanthanide ions with increasing temperature are, among others, associated with the thermally coupled levels (TCLs) of lanthanide ions (e.g., Pr^3+^, Nd^3+^, Ho^3+^, Er^3+^, Tm^3+^) with an energy difference in the range 200 ≤ ΔE ≤ 2000 cm^−1^ [23,32,33,34]. Due to the thermalization processes, some TCLs related to 4f-4f radiative transitions are thermally populated (or depopulated), which results in an increase (or decrease) in their intensity. The obtained intensities of the populated (depopulated) radiative transitions are used to calculate the LIR of the emission bands corresponding to these transitions. As the temperature is determined based on the ratio of the intensity of the emission bands, this method is highly robust to environmental interferences such as fluctuations in pumping intensity or spectrum loss [35]. However, to the best of our knowledge, there are no studies showing that a cellulose-based sensor using luminescent thermometry to determine temperature can be used in the area of health monitoring devices. Therefore, in this research, we focus on the development of a new-generation innovative flexible optical temperature sensor based on regenerated cellulose fibers.

The research carried out in this work allowed us to verify the research hypotheses concerning: (i) modification of cellulose fibers with appropriate luminescence upconversion nanoparticles (NPs) in order to obtain an optical sensor using the luminescence intensity ratio method to determine the temperature; (ii) demonstrating that the optical sensor based on cellulose composite fibers with upconversion NPs has a higher sensitivity and better temperature resolution than other types of cellulose-based temperature sensors presented in the literature so far, and is resistant to EMI; (iii) showing that the obtained cellulose-based composite fibers can be used to produce a wearable sensor to monitor the temperature of the human body.

To prove the validity of these hypotheses, cellulose fibers were prepared according to the dry–wet spinning process, which allows the incorporation of NPs of the modifier into the internal structure of cellulose fibers. This ensures the durability of the prepared fibers during washing, as shown in our previous research [10]. Yttrium fluoride NPs doped with 20% (molar ratio) ytterbium and 2% erbium (YF_3_: 20% Yb^3+^, 2% Er^3+^) were used as thermosensitive upconversion NPs that show bright green emission in near-infrared (NIR) laser excitation. The selection of the YF_3_: Yb^3+^, Er^3+^ system was motivated by two factors: (i) YF_3_ was chosen as the host matrix, because it shows a low phonon energy (~350 cm^−1^) and wide band gap, which limits multiphonon-quenching processes, which are undesirable in luminescent systems [36,37,38,39]; (ii) Er^3+^ ions were selected as activator ions due to their intense green emissions and appropriate energy gap (800 cm^−1^) between two TCLs, while Yb^3+^ ions were used as sensitizer ions, due to their high absorption cross-section in the NIR range (in contrast to Er^3+^ ions) and good match of energy levels with Er^3+^ ions, which ensure efficient energy transfer upconversion (ETU) processes [36,38]. Upconverting NPs were responsible for providing a remote ratiometric temperature reading, while the cellulose fiber served as a flexible platform for these NPs. Then, the influence of the temperature in the range of 298–358 K (25–85 °C) on the spectroscopic properties (including the luminescence intensity ratio (LIR), relative sensitivity, and temperature resolution) of the obtained composite fibers was investigated. Moreover, the resulting composite fibers were processed into a knitted fabric that was tested for use as a wearable temperature sensor.

## 2. Materials and Methods

### 2.1. Materials

Rare-earth (RE) oxides (RE = Y^3+^, Yb^3+^, and Er^3+^) (99.99%, Stanford Materials, Stanford, CA, USA) were dissolved separately in nitric acid (HNO_3_; 65%, ultrapure, POCh. S.A., Gliwice, Poland) to synthesize corresponding RE nitrates. Afterward, the excess of acid was removed by evaporation. Ammonium fluoride (NH_4_F; ≥98 pure p.a., POCh. S.A., GliwicePoland) and PEG 6000 (Sigma-Aldrich, Darmstadt, Germany) were used as received, without further purification. Deionized water was used in all experiments. PLACETAE pulp (polymerization degree $\bar{DP}$ = 1250, 96.8 wt.% of α cellulose from Rayonier (Wildlight, FL, USA) and a 50% aqueous solution of N-methylmorpholine N-oxide-NMMO (from Huntsman Holland BV, Rotterdam, The Netherlands) were used to prepare cellulose solution. Propyl ester of gallic acid-Tenox PG^®^ (purchased from Aldrich, Gillingham, Dorset, UK) was used as an antioxidant to stabilize the molecular weight.

### 2.2. Instrumentations

The cellulose solution was made using a laboratory-scale IKAVISC kneader type MKD 0.6-H60 (Monachium, Germany). The fibers were spun using the dry–wet spinning method on a laboratory-scale piston-spinning device with a spinneret equipped with 18 orifices of 0.4 mm diameter. The powder X-ray diffraction pattern (XRD) was measured with a Bruker AXS D8 Advance diffractometer (Billerica, MA, USA) in Bragg–Brentano geometry, using Cu Kα1 radiation (λ = 1.5406 Å). Transmission electron microscopy (TEM) observations were carried using a Hitachi HT7700 microscope (Tokyo, Japan), applying an acceleration voltage of 100 kV. The FEI Quanta 250 FEG scanning electron microscope equipped with an EDAX detector was used to acquire scanning electron microscopy and EDX images. The mechanical properties of the fibers were measured using a Zwick Z2.5/TN1S tensile testing machine (Ulm, Germany), according to PN-EN ISO 5079:1999. The linear density of the fibers was measured in accordance with PN-EN ISO 1973:1995. The knitted fabric was made on a circular machine characterized by the following technical parameters: cylinder diameter = 4 inches, needle gauge = E14, number of needles = 169. For the knitted fabric produced as part of this work, the following parameters were determined: the number of wales and courses per unit of length according to the standard EN 14971:2006, thickness according to the standard EN ISO 5084:1996, and mass per unit area according to the standard PN-P-04613:1997. The content of the modifier in the cellulose fibers was analyzed by an inductively coupled plasma optical emission spectrometer (ICP-OES Spectro Blue TI, Kleve, Germany). Emission spectra were collected with an Andor Shamrock 500i spectrometer (Belfast, UK) connected to the Peltier-cooled Andor Indus (silicon) CCD camera. The sample was excited by the use of a fiber-coupled, solid-state diode-pumped (SSDP) laser FC-975-2W (CNI, Changchun,, China). In the case of measurements involving YF_3_-modified fibers, the laser beam was focused to about a 0.5 mm spot size and the applied power was adjusted to 0.70 W, which corresponds to a power density of about 360 W/cm^2^. Before acquiring the thermometric experiments, a sufficiently low power of the laser was adjusted to avoid an uncontrolled increase in temperature of the sample, by heating the material with a focused laser beam (the effect of laser-induced heating does not occur when the band intensity ratio associated with the Er^3+^ thermalized levels remains constant). In the case of measurement performed with the use of knitted fabric, the laser beam was focused to an approximately 4 mm size beam spot, and the applied power was adjusted to 0.5 W, which corresponds to a power density of about 4 W/cm^2^. The power density in the applied range (from ~4 to 360 W/cm^2^) enables measurements without heating of the sample and does not influence the temperature sensitivity of the thermometer (because the laser power does not influence the LIR of the bands that are associated with TCLs [27]).

### 2.3. Synthesis of Upconversion Modifier

YF_3_ NPs doped with 20 mol% Yb^3+^ and 2 mol% Er^3+^ were synthesized following our previously reported procedure [27]. To prepare 0.5 g of product, 4.75 mL of 0.5 M YCl_3_ and 1.22 mL of 0.5 M YbCl_3_ were mixed with 1.22 mL of 0.05 M ErCl_3_ aqueous solutions and filled up to 25 mL with water. Next, 25 mL of ethanol was added to the solution of RE^3+^ ions and 0.3 g of PEG 6000 (anti-agglomeration agent,) was dissolved in the as-obtained solution. The solution containing the source of fluoride ions was obtained by dissolving 1.5035 g of NH_4_F (50% molar excess) and 0.25 g of PEG 6000 in 25 mL of water and ethanol in a 12.5/12.5 solvent system. Both solutions were heated up to 323 K. Afterward, the RE^3+^ solution was added drop by drop to continuously stir and heat the solution of NH_4_F to precipitate the lanthanide salt. After the addition, the solutions were stirred for 1 h at 323 K. The pH of the final mixture was adjusted to 2. The as-prepared transparent solution was transferred into a 50 mL Teflon-lined vessel and hydrothermally treated for 16 h at 453 K. After completion of the reaction, the resulting white precipitate was purified by centrifugation and rinsed several times with water and ethanol. The final product (YF_3_: Yb^3+^, Er^3+^) was dried overnight in an oven (358 K), and after that, the sample was ground in an agate mortar. In the next step, it was calcined in a furnace for 4 h at 573 K, in order to improve the crystallinity and luminescence of the material. The sample was then ground again in an agate mortar and dispersed in water.

### 2.4. Preparation of Cellulose Fibers

**Figure 1:**
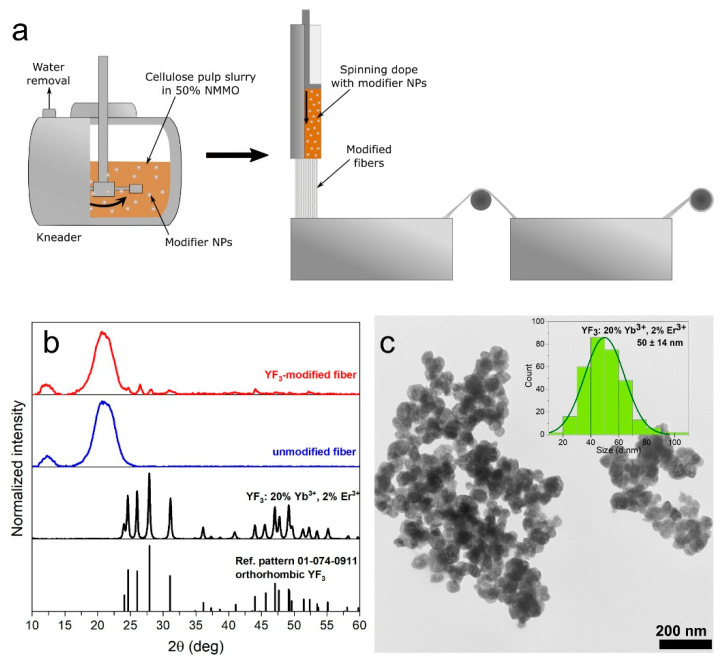
(**a**) Scheme of the formation of the upconversion cellulose fibers; (**b**) powder XRD patterns of modifier NPs, and modified and unmodified cellulose fibers; (**c**) TEM image of YF_3_ NPs; inset shows size distribution of NPs.

A cellulose solution (spinning dope) was prepared by mixing cellulose pulp, aqueous solution of NMMO, antioxidant, and the upconversion modifier in a kneader equipped with a heating system. An aqueous colloidal solution of YF_3_: 20% Yb^3+^, 2% Er^3+^ was added to reach a concentration of about 3.5 wt.% in the final (dry) cellulose fibers. The cellulose was dissolved under low-pressure (≈20 hPa), at temperature not exceeding 115 °C (388 K). During the process, water was removed until its contents in the mixture did not exceed 14 wt.%. The obtained amber-like colored spinning dope was placed into a cylinder (pre-heated to a temperature not exceeding 115 °C (388 K)) and pressed through nozzle holes and the air gap, and it was finally immersed in an aqueous solidifying bath at 20 °C (293 K). The fibers were spun at a take-up speed of 50 m/min, washed, and dried at room temperature. A scheme of the formation of modified cellulose fibers is shown in Figure 1a. The obtained fibers (yarn) were used to produce a single jersey knitted fabric. The stitch of the knitted fabric is shown in Figure S1.

## 3. Results

### 3.1. Structure and Morphology

The synthesized YF_3_ NPs working as a modifier of the fibers show a pure orthorhombic structure matching the reference pattern no. 01-074-0911 from the International Center for Diffraction Data (Figure 1b). The absence of additional XRD lines indicates a pure structure without any impurities. The observed broadening of the reflexes of YF_3_ results from the nanocrystallinity of the obtained particles. The XRD pattern of the modified fibers also confirms the presence of YF_3_ in the fibers matrix. The very low intensity of reflexes associated with YF_3_ in the cellulose matrix is due to the low content of the modifier NPs in the fibers (≈3.5 wt.%). An additional small peak at 2θ values of ~12.3° and an intense broad peak at 2θ~20.3° observed in the XRD of modified fibers correspond to the (hkl 110) and (020) planes of cellulose II [40,41,42].

According to the TEM analysis (Figure 1c), the modifier NPs are in the form of nearly spherical, agglomerated nanoparticles. The average grain size of YF_3_ NPs, calculated based on TEM images, is 50 ± 14 nm (Figure 1c inset). DLS measurements show that the hydrodynamic diameter of YF_3_ NPs (in the form of an aqueous colloid) is in the size range 100–220 nm (Figure S2, ESI†). These two analyses show that YF_3_ NPs are present in water as agglomerates of approximately 2–5 NPs, which indicates that they are suitable for modifying cellulose fibers (as their size is far below the diameter of the fibers).

**Figure 2:**
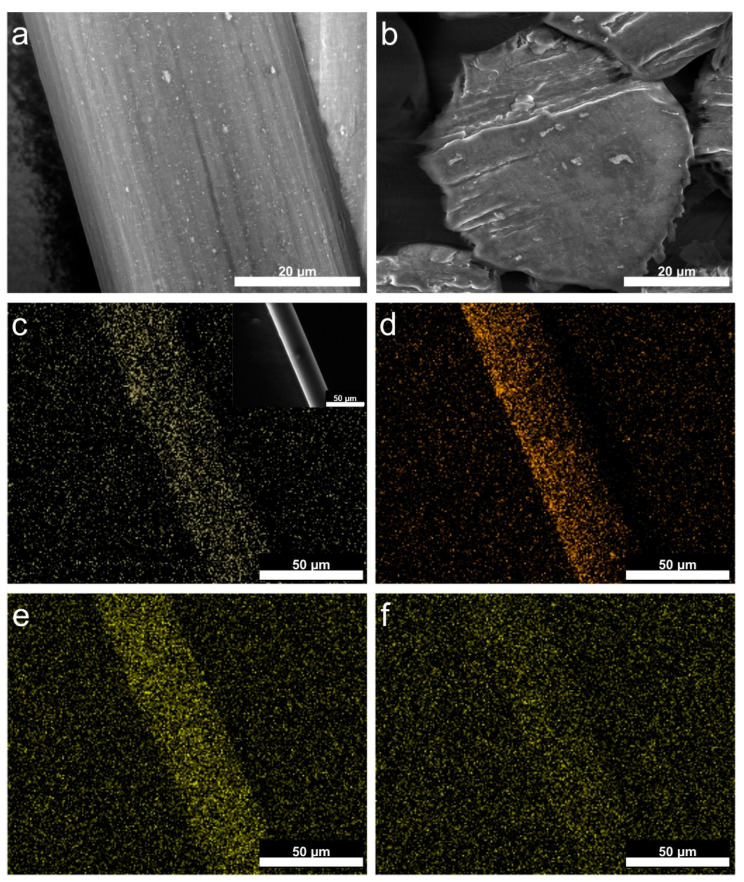
SEM-EDX analysis: (**a**) SEM images of the fibers surface and (**b**) cross-section; (**c**) EDX-mapping of yttrium, (**d**) fluoride, (**e**) ytterbium, and (**f**) erbium; inset presents the field of view.

Such a morphology of the obtained NPs ensures their homogenous distribution within the cellulose matrix, which is confirmed by SEM measurements. The SEM images presented in Figure 2a,b show that YF_3_ NPs (white spots) are uniformly distributed on the surface and in the volume of the fibers, and they do not destroy the final form of the fiber. SEM-EDX mapping (Figure 2c–f) and EDX spectra (Figure S3, ESI†) further prove the presence of elements occurring in the modifier (Y, F, Yb, and Er) within the polymer matrix. These results indicate that YF_3_ NPs are suitable for the modification of cellulose fibers from a technological point of view.

### 3.2. Mechanical Properties

In addition, the mechanical properties of YF_3_-modified fibers (linear density, elastic modulus, tenacity, and elongation at break) are determined and compared with those of unmodified fibers to evaluate the effect of NPs on fiber performance. The results presented in Table 1 show that the tenacity and elongation at break of the YF_3_-modified fibers are slightly reduced, which may stem from the partial loss of elasticity caused by the presence of the inorganic modifier. This is plausibly associated with an increase in the elastic modulus and a decrease in elongation at break. As expected, the linear density increases with the addition of YF_3_ NPs. It should be emphasized, however, that despite the partial weakening of the mechanical performance of the YF_3_-modified fibers, they are still strong enough to be processed into a knitted fabric. Furthermore, the basic structural and physical parameters of the obtained knitted fabric are determined and presented in Table S1, (see ESI†).

### 3.3. Luminescence Properties

**Figure 3:**
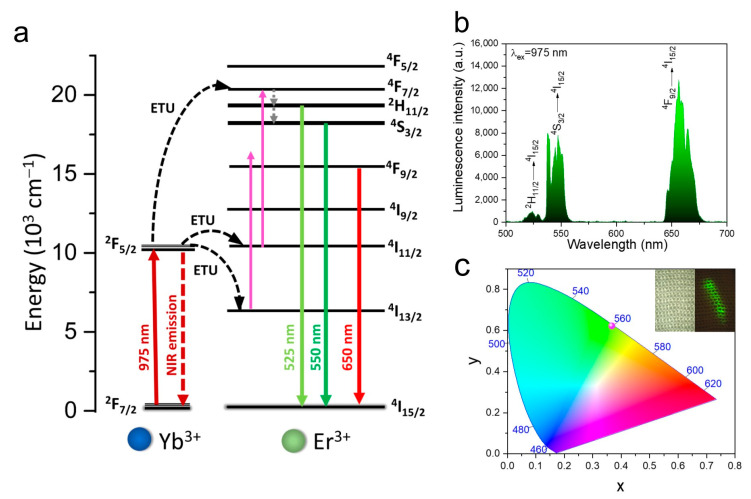
(**a**) Scheme of the energy level diagram of Yb^3+^ and Er^3+^, showing possible upconversion processes in the YF_3_: Yb^3+^, Er^3+^ system, (**b**) upconversion emission spectra of YF_3_-modified fibers, and (**c**) the corresponding chromaticity diagram; the inset shows photographs of a knitted fabric made of YF_3_-modified fibers in daylight (left) and under NIR (λ_ex_ = 975 nm) laser irradiation (right).

The spectroscopic properties of the fibers modified with YF_3_: Yb^3+^, Er^3+^ NPs are analyzed based on the upconversion (UC) emission spectra, recorded at λ_ex_ = 975 nm. In this system, Yb^3+^ ions act as sensitizers (light-harvesting ions), which absorb near-infrared (NIR) energy and transfer it to the nearest emitting Er^3+^ activator ions via the energy transfer upconversion (ETU) mechanism (Figure 3a). The emission spectra of the modified fibers shown in Figure 3b consist of three characteristic, narrow bands from Er^3+^ associated with their 4f-4f radiative transitions, i.e., ^2^H_11/2_→^4^I_15/2_ (525 nm), ^4^S_3/2_→^4^I_15/2_ (550 nm), and ^4^F_9/2_→^4^I_15/2_ (650 nm). The effective color of the luminescence of the YF_3_-modified fibers is determined using the chromaticity coordinates (CIE 1931) calculated based on the recorded emission spectra and it is established as greenish (Figure 3c). This result is consistent with the bright green emission of a knitted fabric (made of YF_3_-modified fibers) excited by NIR irradiation observed with the naked eye (inset of the Figure 3c).

**Figure 4:**
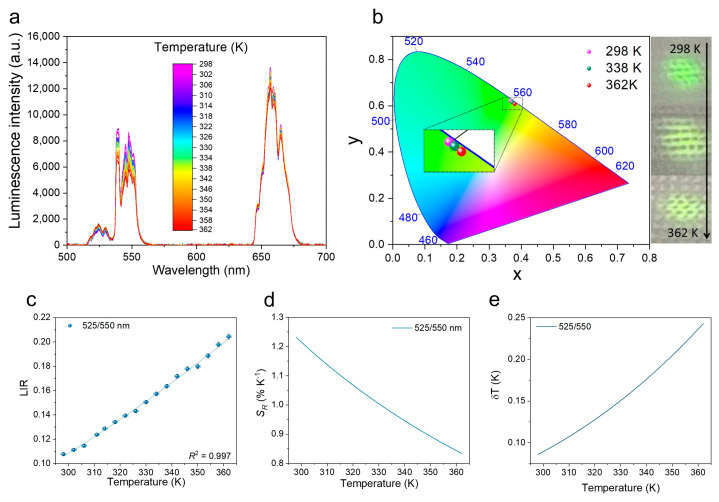
Spectroscopic properties of YF_3_-modified fibers (λ_ex_=975 nm): (**a**) upconversion emission spectra measured as a function of temperature; (**b**) CIE diagram showing the emission color of YF_3_-modified fibers with increasing temperature values (left) and corresponding photographs of a knitted fabric made of YF_3_-modified fibers NIR excitation (right); (**c**) luminescence intensity ratios, corresponding to (**d**) the relative sensitivities *S_r_* and (**e**) the temperature resolution Δ*T*.

In order to investigate the thermometric properties of the obtained fibers, their upconversion emission spectra are measured as a function of temperature, in the range of ≈300–360 K (λ_ex_ = 975 nm). Figure 4a clearly shows that as the temperature increases, the intensity of the band located around 525 nm (Er^3+^: ^2^H_11/2_→^4^I_15/2_) increases and, at the same time, the intensity of the band located around 550 nm (Er^3+^: ^4^S_3/2_→^4^I_15/2_) decreases significantly. This effect is due to the fact that these two transitions originate from the thermally coupled levels (TCLs) of Er^3+^ (^2^H_11/2_ and ^4^S_3/2_) with an energy difference of ΔE = 865 cm^−1^ (derived from the spectrum). In other words, with increasing temperature, the electrons from the lower-energy level (^4^S_3/2_) are transferred to the higher energy level (^2^H_11/2_), thus increasing the population of this level. Due to this, the intensity of the band associated with the higher energy level (^2^H_11/2_) increases with temperature. Thermalization processes induced by the increase in the temperature cause a change in the relative intensity of the ^2^H_11/2_ →^4^I_15/2_ and ^4^S_3/2_ →^4^I_15/2_ transitions, according to the Boltzmann distribution:

(1) $$\mathrm{LIR}=\frac{I_{2}}{I_{1}}=Bexp\left(-\frac{\Delta E}{k_{B}T}\right),$$

where LIR is the luminescence intensity ratio; *I_2_* and *I_1_* refer to the integrated intensity of higher-energy (525 nm) and lower-energy (550 nm) bands, respectively; ∆*E* is the energy difference between the centroids of these two TCLs (the determined Δ*E* value from fitting is 756 cm^−1^, which is similar to the value determined from the spectra); *k_B_* is the Boltzmann constant; *T* is absolute temperature; and *B* is a coefficient depending on the degeneracy of states, the rates of total spontaneous emission, the transition’s branching ratio with respect to the ground state, and the transition’s angular frequency [23,43]. Please note that to determine the band intensity ratio and Δ*E* values, the emission spectra are converted to an energy scale (eV) via the Jacobian transformation [44,45]. However, for the readers’ convenience, all spectra in the manuscript are presented on the wavelength scale. As shown in Figure 4b, with the increase in the temperature from 298 to 362 K, there is a yellow shift in the emission color of the YF_3_-modified fibers (and thus the knitted fabric made of them).

The LIR values calculated using Equation (1) are correlated with temperature, showing a perfect fit, R^2^ > 0.99 (Figure 4c). However, to evaluate the performance of the obtained fibers as an optical thermometer, it is necessary to calculate their relative temperature sensitivity (*S_r_*). Please note that this parameter is independent of the nature of the thermometer and the experimental setup and allows quantitative comparison of the performance of different optical thermometers. *S_r_* expresses the relative change in the ratio of the band intensity per 1 K change in absolute temperature. *S_r_* is defined as:

(2) $$S_{r}=100\%\times \frac{1}{\mathrm{LIR}}\frac{d\mathrm{LIR}}{dT},$$

Based on the fitted LIR values and Equation (2), the *S_r_* values of YF_3_-modified fibers as a function of temperature are determined. *S_r_* values are strongly dependent on the temperature and decrease with the temperature for the 525/550 nm band ratio from 1.23% K^−1^ (at 298 K) to 0.83% K^−1^ (at 362 K). It should be emphasized that this is one of the highest *S_r_* values (around 300 K) for the Er^3+^: 525/550 nm band ratio [46].

Additionally, the temperature uncertainties (Δ*T*; temperature resolutions) are determined based on Equation (3), i.e.,

(3) $$\delta T=\frac{1}{S_{r}}\frac{\delta \mathrm{LIR}}{\mathrm{LIR}},$$

where δLIR is the uncertainty in the determination of the luminescence intensity ratio. The calculated resolution of the optical temperature determination for the Er^3+^ 525/550 nm band intensity ratio at 298 K is about 0.09 K and increases to 0.24 K at 362 K. The calculated *S_r_* and Δ*T* as a function of temperature, together with the corresponding LIR values, are presented in Figure 4c–e.

Furthermore, a thermal cycling of YF_3_-modified fibers is carried out (Figure S4) to verify the applicability and reliability of the developed fiber-thermometer. The LIR values change reversibly with temperature, confirming the thermal stability of the obtained fibers, and proving that they retain their thermometric properties after multiple heating–cooling cycles. Please note that we do not observe any changes in the signal intensities after the performance of thermometric measurements and the performed cycling. Good thermal stability of the prepared materials is ensured by the combination of YF_3_ (stable up to 500 °C [47,48]) with regenerated cellulose fibers (stable up to 300 °C [49,50]), which are stable much above the operating temperature range.

**Figure 5:**
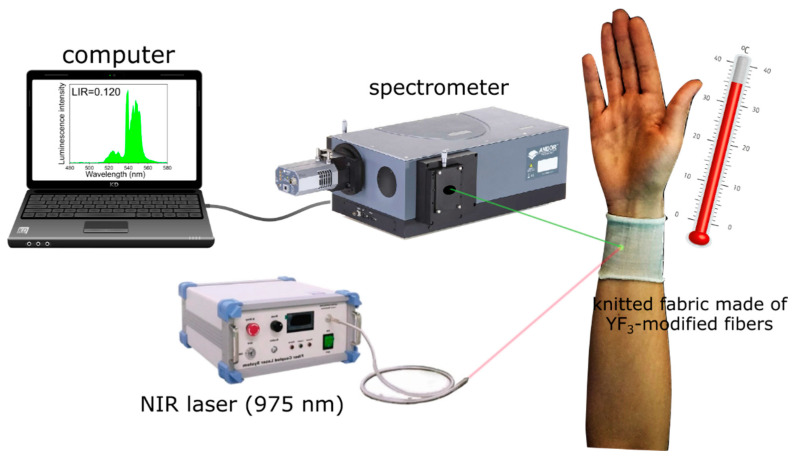
Optical setup for temperature sensing based on a knitted fabric made of YF_3_-modified fibers.

In order to demonstrate the potential use of modified fibers as a wearable temperature sensor, a knitted fabric made of these fibers is used to determine the temperature of human skin. The measurement is carried out on the inner side of the volunteer’s forearm wrapped in the knitted fabric (Figure 5). Based on the obtained LIR value (LIR = 0.120), the forearm skin temperature is measured to be 35.3 °C, which is consistent with the temperature measured with a traditional thermometer. Please note that temperature of the forearm skin is lower than the axillary (underarm) or oral temperature (~36.6 °C) [51]. It should be emphasized that, in this case, the applied laser power density is significantly lower than in the case of the YF_3_-modified fibers, which proves that it is possible to obtain reliable results even with the use of such low laser power densities, which is a very important factor in the case of real applications, e.g., biological or industrial processes, where the use of high-power lasers is usual.

Table 2 compares various examples of cellulose-based temperature sensors in terms of their performance with the optical sensor proposed in this work. The developed YF_3_-modified cellulose fibers exhibit various advantages, such as higher relative sensitivity and better temperature resolution. Moreover, the sensor presented in this research is resistant to EMI, unlike resistive or capacitive-type sensors.

## 4. Conclusions

In summary, we designed a flexible optical temperature sensor based on regenerated cellulose fibers, modified with upconversion NPs. The performed studies proved its effectiveness in use and confirmed the validity of our research hypotheses. Namely, incorporation of YF_3_: 20% Yb^3+^, 2% Er^3+^ upconversion NPs (optimal composition) into cellulose fibers assured a thermally sensitive emission under NIR excitation, while cellulose fibers served as a flexible carrier for upconversion NPs. The temperature measurement was based on the determination of the ratio of the intensity of emission bands associated with transitions from thermally coupled levels of Er^3+^, i.e., ^2^H_11/2_ →^4^I_15/2_ and ^4^S_3/2_ →^4^I_15/2_. Due to the fact that the presented temperature sensor is ratiometric, and therefore does not rely on a direct change in the measured parameter, it is robust to environmental interferences. Other benefits of this upconversion temperature sensor are its outstanding relative sensitivity and temperature resolution (*S_r_* = 1.232% K^−1^ and Δ*T* = 0.09 K, both at 298 K), which allow very precise temperature sensing in the range of 298–362 K. Importantly, this sensor shows constant values of the parameters discussed in the paper during repeated measurements in the heating–cooling cycles, which indicates its high reliability and stability. Furthermore, as the principle of operation of the discussed sensor is based on the measurement of emission spectra, and the sensor itself does not conduct electricity, it is inherently immune to electromagnetic interferences. The developed thermometer might be especially useful in the new generation of wearable devices for human health monitoring.

## Figures and Tables

**Table 1:** Mechanical properties of the fibers.

	Unmodified Cellulose Fibers	YF_3_-Modified Cellulose Fibers
Elastic modulus [cN/tex]	1218	1519
Tenacity [cN/tex]	28.7	26.2
Elongation at break [%]	9.4	8.0
Linear density [tex]	0.3264	1.450

**Table 2:** Comparison of the cellulose-based temperature sensors reported in the literature with the YF_3_-modified cellulose fibers shown in this work.

Material	Type of Sensor	Sensing Range [K]	Δ*T* [K]	*S_r_*, [% K^−1^]	Susceptible to EMI	Ref.
YF_3_-modified cellulose fibers	Ratiometric UC	298–362	0.09 (at 298 K)	1.23 (at 289 K)	No	This work
Cellulose-PPy nanocomposite	Capacitive	288–323	n.d.	n.d.	Yes	[4]
PEDOT-PSS-SWCNT coated jute fibers	Resistive	297–308	n.d.	0.23	Yes	[5]
WS_2_-QDs/RGO coated cotton fabric	Resistive	77–398	0.01 (0.06 human trial)	0.56 (298-396 K)	Yes	[22]
Cellulose/RGO composite films	Capacitive	298–353	n.d	n.d	Yes	[52]
HPC-ethylene glycol	Colorimetric	253–298	2	n.a.	No	[53]
CNF/CNT nanohybrid	Resistive	303–353	n.d.	1.081	Yes	[54]

Abbreviations: CNF = cellulose nanofiber, CNT = carbon nanotube, HPC = hydroxypropyl cellulose, PEDOT = poly(3,4-ethylenedioxythiophene), PPy = polypyrrole, PSS = polystyrene sulfonate, RGO = reduced graphene oxide, SWCNT = single-walled carbon nanotubes, QDs = quantum dots.

## Data Availability

All of the relevant data are available from the corresponding authors upon reasonable request. Source data are provided with this paper.

## Supplementary Materials

The following supporting information can be downloaded at: https://www.mdpi.com/article/10.3390/nano12111926/s1, Figure S1: Right side of the knitted fabric; Figure S2: DLS analysis of YF_3_: 20% Yb^3+^, 2% Er^3+^ NPs; Figure S3: EDX spectra of YF_3_-modified cellulose fibers; Table S1: Basic structural and physical parameters of a knitted fabric made of YF_3_-modified cellulose fibers; Figure S4: Thermal cycling of YF_3_-modified fibers between 298 K and 362 K, for the determined thermometric parameters, i.e., Er^3+^: 525/550 nm band ratio, λ_ex_ = 975 nm.
