# Supplementary material for: Ratiometric Upconversion Temperature Sensor Based on Cellulose Fibers Modified with Yttrium Fluoride Nanoparticles
Source: Nanomaterials (Basel). 2022 Jun 4;12(11):1926. doi: 10.3390/nano12111926 (PMC9182498; doi:10.3390/nano12111926)
Supplement: Supplementary file 1 [file nanomaterials-12-01926-s001.zip › nanomaterials-1747698-supplementary.pdf]

Supplementary materials for:

Ratiometric upconversion temperature sensor based on cellulose fibers modified with yttrium fluoride nanoparticles

Małgorzata Skwierczyńska [1,*], Natalia Stopikowska [1], Piotr Kulpiński [2], Magdalena Kłonowska [3], Stefan Lis [1] and Marcin Runowski [1,4]

[1] Adam Mickiewicz University, Faculty of Chemistry, Department of Rare Earths, Uniwersytetu Poznańskiego 8, 61-614 Poznań, Poland, natalia.stopikowska@amu.edu.pl (N.S.); blis@amu.edu.pl (S.L.)
[2] Lodz University of Technology, Faculty of Material Technologies and Textile Design, Department of Mechanical Engineering, Informatics and Chemistry of Polymer Materials, Żeromskiego 116, 90-924 Łódź, Poland, piotr.kulpinski@p.lodz.pl (P.K.)
[3] Lodz University of Technology, Faculty of Material Technologies and Textile Design, Department of Knitting Technology and Textile Machines, Żeromskiego 116, 90-924 Łódź, Poland, magdalena.klonowska@p.lodz.pl (M.K.)
[4] Universidad de La Laguna, Departamento de Física, Apdo. Correos 456, E-38200, San Cristóbal de La Laguna, Santa Cruz de Tenerife, Spain, mrunowsk@ull.edu.es (M.R.)
* Correspondence: malgorzata.skwierczynska@amu.edu.pl; (M.S.)

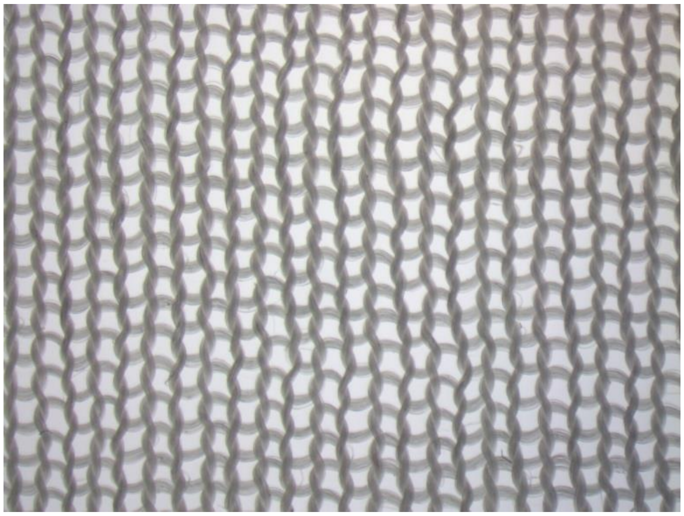

Figure S1 Right side of the knitted fabric

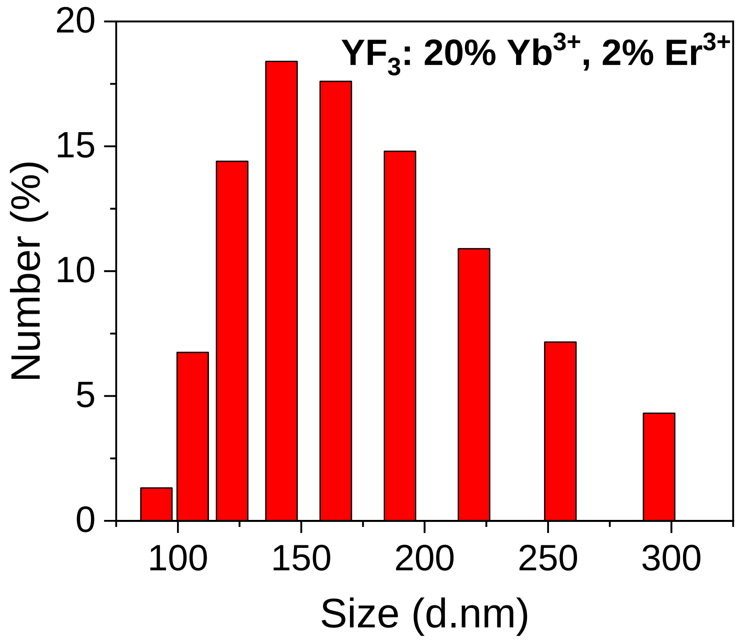

Figure S2 DLS analysis of YF_3: 20 % Yb^{3+}, 2 % Er^{3+} NPs.

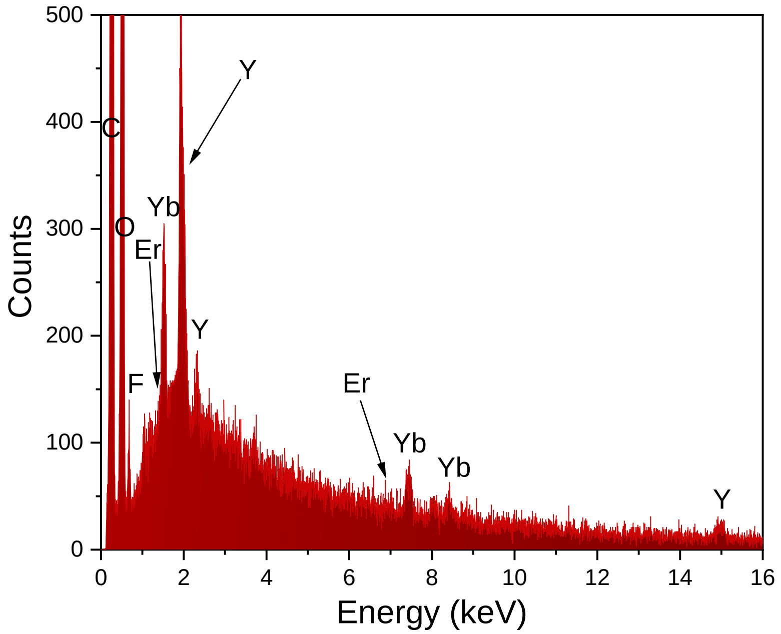

Figure S3 EDX spectra of YF_3-modified cellulose fibers.

Table S1 Basic structural and physical parameters of a knitted fabric made of YF_3-modified cellulose fibers.

	**Number of wales [wale/cm]**	**Number of courses [course/cm]**	**Fabric thickness [mm]**	**Mas per unit area [g/m²]**
Mean value	10.0	14.0	0.67	125.0
Standard deviation	0.52	0.48	0.01	2.57

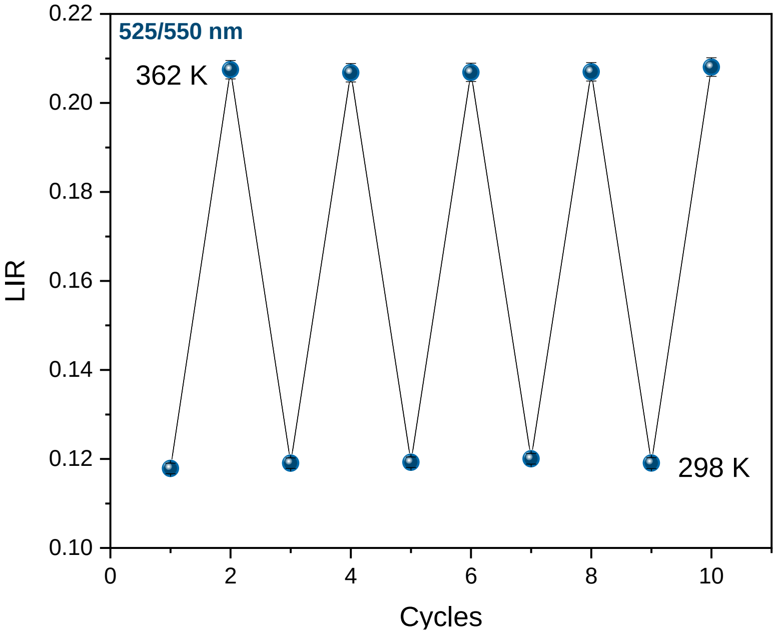

Figure S4 Thermal cycling of YF_3-modified fibers between 298 K and 362 K, for the determined thermometric parameters, i.e. Er^{3+}: 525/550 nm band ratio, λ_{ex} = 975 nm.
